# The Role of HPV in Head and Neck Cancer Stem Cell Formation and Tumorigenesis

**DOI:** 10.3390/cancers8020024

**Published:** 2016-02-19

**Authors:** Mark S. Swanson, Niels Kokot, Uttam K. Sinha

**Affiliations:** Department of Otolaryngology—Head and Neck Surgery, University of Southern California, 1450 San Pablo St, Suite 5100, Los Angeles, CA 90033, USA; Mark.Swanson@med.usc.edu (M.S.S.); Niels.Kokot@med.usc.edu (N.K.)

**Keywords:** cancer stem cell, cancer initiating cell, human papilloma virus, HPV, Notch, Oct-4, Nanog

## Abstract

The cancer stem cell (CSC) theory proposes that a minority of tumor cells are capable of self-replication and tumorigenesis. It is these minority of cells that are responsible for cancer metastasis and recurrence in head and neck squamous cell cancers (HNSCC). Human papilloma virus (HPV)-related cancer of the oropharynx is becoming more prevalent, which makes understanding of the relationship between HPV and CSCs more important than ever. This relationship is critical because CSC behavior can be predicted based on cell surface markers, which makes them a suitable candidate for targeted therapy. New therapies are an exciting opportunity to advance past the stalled outcomes in HNSCC that have plagued patients and clinicians for several decades.

## 1. Introduction

Normal stem cells and malignant cells share several common features. Both are capable of self-replication, heterogenous cell population creation, and perpetuation of these qualities through self-renewal. However, not all malignant cells are capable of tumorigenesis and self-renewal. In fact, only a minority of tumor cells possess these qualities, which gave rise to the cancer stem cell (CSC) theory. These shared features between normal stem cells and CSCs have a molecular basis in several of the pathways used by stem cells for cellular differentiation and replication including Nanog, Oct-4, and Notch, and have been isolated in a variety of malignancies, including oropharyngeal head and neck squamous cell carcinoma (HNSCC). These same pathways are modified by the human papilloma virus (HPV), which makes the CSC theory especially applicable to the rising incidence of HPV-related oropharyngeal HNSCC.

The unique characteristics of CSCs make them a potentially effective target for cancer therapeutics to prevent recurrence and metastasis. Tumors develop metastases by shedding cells into the lymphatic and hematogenous circulation, which seeds distant tissues. Although both CSCs and non-CSC tumor cells are shed, metastatic tumor growth will only occur with the former, making them a potential therapeutic target. Fortunately, HNSCC CSCs behavior can be predicted by cell surface markers, which allows for isolation and targeted therapies.

HPV incidence specifically in oropharyngeal HNSCC is on the rise, and possesses a unique mechanism for tumorigenesis from alcohol and tobacco induced cancers. It will be important to relate our advancement in understanding of CSCs to the growing incidence in HPV related tumors to improve treatment strategies and improve patient outcomes. As such, this paper will review the supporting literature for HPV's role in CSC formation and tumorigenesis.

## 2. The Cancer Stem Cell Theory

Cancers are a genetically and functionally heterogenous group of cells. For example, when isolated cancer cells are extracted from a tumor and re-injected into a recipient host, only a limited group of cells are capable of tumor formation. It is this difference in functional capacity that defines the cancer stem cell theory.

CSCs are a minority sub-population of tumor cells that have capacity for asymmetric cell division, differentiation, and self-replication that can regenerate a complete heterogenous tumor. It is the ability of CSCs to differentiate and replicate that gives them metastatic and tumorigenic potential, while non-CSCs that metastasize ultimately degenerate without formation of a metastatic tumor. They are distinguished from non CSC tumor cells by their function, and can be isolated by unique expression of surface proteins that are often found on normal embryonal or epithelial stem cells.

The existence of CSCs was first examined in acute myelogenous leukemia (AML) where a subpopulation of CD34^+^/CD38^−^ cells had proliferative and self-renewal properties capable of producing the genotypic and functional heterogeneity of the cancer, while other tumor cells could not reproduce viable tumor [1]. Further support of the CSC theory was demonstrated in melanoma with CD271^+^ cells being more tumorigenic than CD271^−^ cells from the same tumor. In this study, CD271^+^ malignant cells were isolated and injected into mice, and formed tumors in 70% of injections compared to only 7% of CD271^−^ cells [2]. These CSCs made up a minority of these tumors ranging from 2.5% to 41% of the tumor cell population. Since only a portion of tumor cells are capable of tumorigenesis, and these cells are measurably different, CSCs became an interesting research target for novel therapeutics.

There are opposing theories about how CSCs are formed. One theory proposes that CSCs develop directly from malignant transformation of normal stem cells. This proposes that normal epithelial stem cells have a long lifespan compared to differentiated cells, which allows for accumulation of genetic mutations [3,4]. Due to the high cellular turnover of the upper aerodigestive tract mucosa, it is likely that the epithelial stem cells are responsible for accumulating the genetic alterations necessary for malignant transformation [3]. The malignant transformation of epithelial stem cells can result in tumor cells harboring the same genetic pathways for self-replication and heterogenous cell population creation as their normal stem cell counterparts. Another proposed theory is that developing or differentiated cells undergo de-differentiation to become CSCs. This theory is supported by evidence that mutations in p53, Oct4, Sox2, Nanog, and KLF4 induce de-differentiation of cells [5,6,7,8].

## 3. Cancer Stem Cells in Head and Neck Cancer

The lack of significant progress in the treatment of advanced HNSCC has demanded the re-visitation of tumor biology to identify better treatment strategies. With mounting evidence for the Cancer Stem Cell theory, researchers have looked to apply this concept to head and neck cancers. The current therapies of surgery and radiation treat whole tumor volumes, which lacks specificity for the CSCs that are responsible for disease recurrence. With identification of CSCs in HNSCC, there is potential for novel treatment strategies, with potential for improved patient outcomes.

### 3.1. Emerging Evidence of HNSCC Cancer Stem Cells

Prince *et al.* provided convincing evidence for the existence of HNSCC CSCs through identification of unique tumorigenic properties of cells expressing CD44 surface antigen and aldehyde dehydrogenase (ALDH) activity [9]. CD44 is a cell-surface protein involved in cellular proliferation and migration that has been previously identified as a marker for breast and prostate CSCs. HNSCC tumors show a proportion of CD44^+^ cells ranging from 0.1% to 41.72% of the tumor population, and when isolated, CD44^+^ cells also share CSC properties [9]. Injection of 1000 HNSCC CSCs that were ALDH^+^CD44^+^ let to tumor development in 13 of 15 mice, while injection of 10,000 non-CSCs which were ALDH^−^CD44^−^ only led to tumor development in 2 of 15 mice [10]. Not only does this support the existence of CSCs in HNSCC, but it also gives a mechanism for isolation of these cells from non-CSCs.

The Cancer Stem Cell theory can also be applied to HNSCC recurrence after resection with negative margins. Field cancerization describes the genetic alterations present in normal appearing cells surrounding the tumor and throughout the aerodigestive tract. Although not frankly malignant, these cells require relatively few genetic hits for malignant transformation, thus harboring potential for cancer recurrence and second primary tumors. CSC markers including ATR, ABCG1, Oct4, and Sox2 have been detected in mucosa adjacent to tumors, suggesting a possible role of CSCs in field cancerization development and tumor recurrence [3,11].

### 3.2. Unique Response to Therapy

Cancer stem cells (CSCs) are increasingly becoming an area of investigation for cancer therapeutics because untreated CSCs are thought to contribute to disease recurrence and metastasis. Current therapies target tumor bulk, but lack specificity for CSCs. Since there is evidence of radiation and cisplatin resistance amongst breast and HNSCC CSCs when compared to the non-tumorigenic cells, there is interest in developing therapies specific to CSCs [12,13,14,15,16,17,18].

Cultured breast cancer CSCs expressing CD44 were more resistant to radiotherapy and had higher Notch-1 activation. This led to a higher proportion of CSCs after radiation treatment, and is thought to explain rapid regrowth of tumors during radiation therapy treatment gaps [13,16]. A postulated etiology of radiotherapy resistance is that breast and HNSCC CSCs have a lower level of reactive oxygen species (ROS) and more efficient free radical scavengers. Radiotherapy primarily exerts its effect through oxygen ionization causing DNA damage, so more efficient ROS scavenging of CSCs can result in decreased treatment efficacy. Radiotherapy resistance is also seen in HNSCC CSCs due to reduced ROS resulting in less DNA damage after radiotherapy [15].

Similar findings of CD44^+^ breast cancer cells show a chemoresistance as well [14]. In this study, core biopsies were cultured before and after 12 weeks of neoadjuvant chemotherapy. After treatment, a higher proportion of CD44^+^ cells survived, and had increased tumorigenic efficiency, indicating a resistance to therapy by CSCs. This chemoresistance may be induced by resistance to apoptosis and expression of drug efflux pumps [19], or by over expression of ALDH by CSCs which protects against the effects of cisplatin [20]. Cisplatin chemoresistance has also been found in oral HNSCC CSCs expressing CD133 and CD10. CD133^+^ cells show higher cisplatin chemoresistance than the majority of the CD133^−^ tumor cell population, but when CD133 expression was suppressed using a viral vector, the chemoresistance was reduced [21]. A similar trend for CD10^+^ HNSCC CSCs, which are more cisplatin and radioresistant than their CD10^−^ counterparts [22].

Another study on HNSCC evaluated the proportion of CSCs in a tumor and evaluated radiotherapy outcomes. They propose that during radiation therapy, susceptible tumor cells are killed and replenished with radioresistant CSCs. Tumors with elevated CSCs had higher rates of local recurrence, distant metastases and reduced overall survival after radiation [23]. HNSCC appears to follow trends with other malignancies that CSCs are inherently resistant to standard therapies and require development of new treatments to improve outcomes.

### 3.3. Stem Cell Pathways for Tumorigenesis

There are molecular pathways and cell surface markers that are specific for stem cells, owing to their unique function. Sox2, Oct-1, Oct-4, and Nanog gene expression is unique to stem cells, because they regulate pleuripotency [24,25,26]. Cancer cells can utilize normal stem cell pathways to avoid apoptosis and preserve stemness to allow for tumorigenesis. This has been demonstrated in viruses such as HBV which utilizes the Oct-4 and Nanog pathways to maintain stemness in the pathogenesis of hepatocellular carcinoma [27]. However altered stem cell pathways do not work in isolation, but rather have significant interactions to promote tumorigenesis and epithelial-mesenchymal transition. There is a complicated integration of signaling pathways which results in the CSC phenotype, and many different pathways can converge to a common downstream effect [28]. Discussed below is a review of some stem cell pathways that are common to HNSCC CSCs.

#### 3.3.1. Nanog

Nanog is a key protein in stem cell regulation that is frequently aberrant in malignancies. It is a transcription factor that facilitates self-renewal of embryonic stem cells though maintenance of pleuripotency. Stem cells have high Nanog expression, and as cells differentiate, expression is quickly suppressed. This process is unregulated in malignancies, and is over expressed to maintain CSC characteristics. In a normally functioning cell, p53 binds to the Nanog gene promoter to suppress its expression [29]. The suppression of Nanog by p53 promotes differentiation and allows for apoptosis. HPV causes p53 degradation through its E6 protein, thus leading to de-inhibition of Nanog and promotion of cell stemness.

#### 3.3.2. Notch

The Notch pathway is elicited by normal stem cells for cellular differentiation. The Notch genes encode a transmembrane receptor, which when activated, promotes exiting the cell cycle and allowing cellular differentiation of keratinocytes [30,31]. The Notch pathway also plays a role in tumorigenesis and has an anti-apoptotic effect in many tumors [32]. HPV^+^ tumors have an altered expression of Notch, which promotes cell propagation and CSC development [30,32,33].

#### 3.3.3. Oct-4

Oct-4 is another transcription factor that is necessary for stem cell self-renewal. This transcription factor is regulated by the POU family of genes, and becomes down-regulated as cells differentiate. Oct-4 is expressed by pluripotent cells and not by differentiated cells, it has been validated as a marker for stem cells in germ cell tumors [34].

Tumor cells with Oct-4 expression correlate with CSC behavior, tumorigenic potential, and aggressive clinical features such as metastasis, disease progression, and reduced survival in bladder and breast cancer [35,36,37]. In these studies, Oct-4 expression was also seen to be higher in ALDH positive tumor cells, which is another known HNSCC CSC tumor marker. When examining HNSCC lymph node metastasis in a human-in-mouse model, metastasized cells over-expressed CSC markers including ALDH and Oct-4 [38].

### 3.4. Cancer Stem Cellular Markers

Just as in hematologic, CNS, and other epithelial malignancies, HNSCC tumor cell behavior can be predicted based on cell surface markers [39,40,41,42,43,44,45,46]. Cellular markers such as CD44, ALDH, CD133, CD10, CD98, and CD24 identify cells which have CSC properties in HNSCC. Distinguishing these cells capable of tumorigenesis allows for distinction from other cells for possible development of targeted treatments.

In addition to being a CSC marker, CD44^+^ cells were identified as CSCs in breast cancer, and were later discovered to have similar properties in HNSCC [9]. CD44^+^ HNSCC cells correlate with more aggressive features and higher metastatic potential [47]. The CD44^+^ HNSCC cells share similar histologic features to stem cells, while CD44^−^ cells have histology more similar to differentiated cells. Like normal stem cells, CD44^+^ cells express BMI1, which is involved with self-renewal, and stain with Cytokeratin 5/14—a stem cell marker [9]. As such, injection of CD44^+^ HNSCC tumor cells produced *in vivo* tumors in 20 of 31 injections while CD44^−^ cells only produced a tumor in 1 of 40. The tumors that developed from the CD44^+^ cells had identical histology and possessed a diverse group of CD44^+^ and CD44^−^ cells as seen in the original tumor [9]. Just as normal stem cells divide to give rise to identical stem cells and a diverse group of differentiated cells, CD44^+^ cells can self-replicate in addition to producing the gamet of tumor stromal cells. These experiments demonstrate that CD44 serves as a marker for CSC that express genetic patterns and behavior similar to stem cells, and are unique amongst the majority of cancer cells in their ability for self-replication, differentiation, and tumorigenesis.

Although CD44 serves as a marker for HNSCC CSCs, it is abundant in HNSCC and lacks a high specificity [48]. Aldehyde dehydrogenase (ALDH) is an enzyme found in normal stem cells, and has been identified early as a marker of HNSCC CSCs [18,49]. In a study of 5 HNSCC tumors, CD44^+^ALDH^+^ cells resulted in tumor formation when injected into immunocompromised mice, while CD44^+^ALDH^−^ cells failed to form tumors. In addition, the CD44^+^ALDH^+^ cells had upregulated expression of stem cell genes, Oct-4, Nanog, and Sox2 [18]. In addition, ALDH expression was seen in only 1%–7.8% of HNSCC cells, and were able to form tumors with injections of as few as 500 cells into mice [49]. This indicates that ALDH may serve as more specific CSC marker than CD44.

With growing research into this area, additional markers of CSC behavior in HNSCC have been discovered. In addition to CD44 and ALDH, CD133^+^, CD10^+^, and CD98^+^ cells have CSC behavior in HNSCC [21,22,26]. The diversity of CSC markers highlights the heterogeneity of tumors, and offers several potential therapeutic targets. Antibodies have been developed to bind to these cell surface markers as a more selective treatment of CSCs, which has potential to reduce chemoresistance or recurrence and metastasis rates [21,26].

## 4. Human Papilloma Virus in Oropharyngeal Head and Neck Cancer

HPV related tumors have different genetic alterations and have a different pathway to malignancy than non-HPV related tumors, which results in a tumor with different clinical characteristics. In HNSCC, the strongest association with HPV has been found in oropharyngeal sites, and thus has received the most investigation. A meta-analysis of HPV related oropharyngeal HNSCC reviewing 18 studies showed better clinical outcomes including higher overall survival, better disease free survival, and lower rates of local recurrence for HPV related tumors compared to non-HPV related tumors [50]. Evidence has become substantial for the prognosis benefit in HPV related oropharyngeal HNSCC, but is less clear for non-oropharyngeal head and neck subsites. In addition to the oropharynx, HPV has less frequently been detected in tumors of the hypopharynx, larynx, and oral cavity [51]. The data is not as robust, but in one prospective study there was no survival or recurrence benefit for HPV related tumors in non-oropharyngeal sites [51].

### 4.1. Relationship of HPV with Cancer Stem Cells

CSCs share much of the genetic machinery as normal stem cells to maintain pleuripotency and allow for self-replication and tumor formation. HPV infects the basal layer of epithelial cells and specifically alters pleuripotency genes. Malignant transformation of stem cells retains many of the stem cell molecular pathways, which provides a mechanism for CSC formation unique to HPV^+^ HNSCC tumors when compared to sporadic HNSCC.

HPV related oropharyngeal HNSCC is rising in incidence, and there are early investigations to suggest that CSCs play a significant role in carcinogenesis. In a small study of six oropharyngeal HNSCC tumor specimens, HPV^+^ tumors had a higher proportion of CSCs compared to HPV^−^ tumors [52]. In this study, HPV^+^ and HPV^−^ oropharyngeal tumors were evaluated for CSC density based on CSC marker expression (ALDH1) and tumor sphere formation. The HPV^+^ tumors had a 62.5-fold greater proportion of CSCs, which was attributed to p53 inactivation by HPV. Compared with HPV^−^ tumors, HPV^+^ tumors have a higher proportion CSCs and have a 2-fold increased tumorigenicity. Additionally, a study of four HPV^−^ oral and oropharyngeal HNSCC cell lines infected with the HPV genome resulted in tumors with increased growth and self-renewal capacity [53]. Additionally, these HPV infected tumors had over expression of ALDH and increased cellular invasion and migration. It seems counterintuitive to see a better prognosis in HPV^+^ tumors despite an increased proportion of CSCs, which have been shown to have radiation and chemotherapy resistance. This suggests that CSCs are not homogenous between tumors, and that their phenotype is more important in clinical outcome than their number [52]. These findings are on small sample sizes and are not definitive, but may suggest a relationship between HPV and CSC formation. In fact, in another conflicting study, HPV^+^ tumors did not have higher expression of ALDH compared to HPV^−^ tumors [54], and a mouse model of HPV^+^ HNSCC did not show a higher proportion of CSCs than HPV^−^ HNSCC [47]. The relationship is still poorly understood and requires additional research.

### 4.2. HPVs Mechanism for Oncogenesis and CSC Formation

Cancer induced by viruses has long been a subject of study. In fact, viruses including Hepatis B, Hepatitis C, HPV, Epstein-Barr, and others are thought to cause 20% of the world's cancer by dysregulation the cell cycle, altering DNA, and inhibiting apoptosis [55,56]. HPV is carcinogenic in part due to E6 and E7 proteins causing dysregulation of p53 and Rb control of apoptosis and cell cycle regulation [57,58].

HPV deregulates p53 though the E6 protein, which is an important cellular pathway in maintaining stemness. P53 is responsible for cessation of cell growth and promotion of apoptosis in the presence of cellular damage, and binds to gene promoter regions to alter Nanog and Notch expression [29]. This is demonstrated when p53 is deleted in breast tissue, the epithelium has a higher proportion of stem cells with high regenerative capacity and altered Notch signaling [59]. P53 normally inhibits the notch pathway, but when infected with HPV this inhibition is lifted, causing a more proliferative state through activated Notch signaling. HPVs dysregulation of p53 through E6 leads to the increased likelihood of CSC production and tumorigenesis. Mutations in the tumor supressor retinoblastoma gene is a common finding in many malignancies, and its inactivation by HPV is a key etiologic factor in tumorigenesis. Activated RB acts to prevent cell cycle progression. It accomplishes this by binding and inactivating the E2F protein, which is a transcription factor which promotes cell division. When a cell approaches cellular division, the RB protein is phosphorylated and inactivated, which releases the E2F protein thus allowing progression from the G1 to the S phase of cell division. Loss of the RB gene predisposes to cell proliferation and oncogenesis. The E7 protein of HPV complexes with RB, which frees RB from E2F and promotes unregulated cell proliferation [60]. E2F motifs are found in the promoter regions of pleuripotency regulators such as Nanog. The release of E2F has shown an increase in expression of stem cell genes, and offers a plausible route for CSC formation, although data specifically relating to HPV^+^ HNSCC is lacking [61].

HPV is a very prevalent virus, and infection alone is not sufficient for malignant degeneration. HPV has also been shown to cause destabilization of chromosome structure with deletions and translocations of chromosomes in immortalized keratinocytes. This destabilization is a mechanism for additive genetic alterations which affect gene expression and malignant potential [62].

HPV can also exert oncogenic pressure through epigenetic changes such as altered methylation and chromatin structural changes to oncogenes. E7 expression causes upregulation of methyltransferase and demethylase proteins in keratinocytes resulting in reduced methylation of H3K27. This demethylation had downstream effects of increased expression of homeobox genes and p16 expression [55]. These changes promote cell stemness through alteration of protein expression, independent of their dysregulation of RB and p53 [63].

E6 and E7 dysregulation of the cell cycle is not sufficient for malignant transformation, and cells require additional genetic and epigenetic changes. One such mutation is in the stem cell Notch pathway, which is seen with reduced expression in cervical cancers [30,32,33]. The Notch1 protein is elevated in premalignant HPV lesions [33,64,65], but at reduced expression in HPV associated cervical cancers, suggesting that it represents an important pathway for HPV oncogenesis [32].

## 5. Conclusions

The cancer stem cell model dictates that only a fraction of tumor cells are capable of tumor propagation, recurrence, and metastasis. These CSCs utilize pathways similar to normal stem cells to maintain pluripotency. HNSCC fits the CSC model, and HPV related oropharyngeal tumors have a unique but poorly understood relationship with CSCs. Importantly CSCs can be isolated with cell surface markers, which allows for research into novel targeted therapy.

HPV^+^ oropharyngeal tumors propagate CSCs differently than HPV^−^ tumors due to the molecular mechanisms of tumorigenesis unique to HPV. HPV preferentially affects keratinocyte stem cells and alters inherent stem cell pathways to promote CSC behavior. This is accomplished through alteration of stem cell pathways p53, Nanog, Notch, and Oct-4. This is seen by elevated Notch expression, disinhibition of Nanog expression, and activated Oct-4 in HPV related tumors. The utilization of these pathways supports the CSC theory in HPV related tumors, and can be an etiology of increased rates of metastasis.

CSCs represent an important therapeutic target for cancer treatment, because they represent the cells capable of tumor recurrence and metastasis. The importance is enhanced due to the problem of increased resistance to conventional treatments of CSCs. Treatment of only non-CSCs would result in tumor recurrence from the surviving CSCs, but elimination of the cell population capable of replication would induce tumor regression. HNSCC CSCs have shown radiation therapy and chemotherapy resistance, and insufficient treatment can lead to fast recurrences with selection for resistant CSCs. Fortunately, there are distinct differences in CSCs and non-CSCs in cell surface markers and activated cellular pathways. It is the differences such as Oct-4, Nanog, and Notch signaling that are potential therapeutic targets. Changing the way cancer is thought of and treated has exciting potential to finally break the hurdle patients and clinicians have been struggling with for several decades and improve patient outcomes and quality of life.

## References

[1] Bonnet D., Dick J.E. (1997). Human acute myeloid leukemia is organized as a hierarchy that originates from a primitive hematopoietic cell. Nat. Med..

[2] Boiko A.D., Razorenova O.V., van de Rijn M., Swetter S.M., Johnson D.L., Ly D.P., Butler P.D., Yang G.P., Joshua B., Kaplan M.J. (2010). Human melanoma-initiating cells express neural crest nerve growth factor receptor CD271. Nature.

[3] Simple M., Suresh A., Das D., Kuriakose M.A. (2015). Cancer stem cells and field cancerization of Oral squamous cell carcinoma. Oral Oncol..

[4] Feller L.L., Khammissa R.R., Kramer B.B., Lemmer J.J. (2013). Oral squamous cell carcinoma in relation to field precancerisation: pathobiology. Cancer Cell Int..

[5] Herreros-Villanueva M., Zhang J.S., Koenig A., Abel E.V., Smyrk T.C., Bamlet W.R., de Narvajas A.A., Gomez T.S., Simeone D.M., Bujanda L. (2013). SOX2 promotes dedifferentiation and imparts stem cell-like features to pancreatic cancer cells. Oncogenesis.

[6] Kumar S.M., Liu S., Lu H., Zhang H., Zhang P.J., Gimotty P.A., Guerra M., Guo W., Xu X. (2012). Acquired cancer stem cell phenotypes through Oct4-mediated dedifferentiation. Oncogene.

[7] Moon J.H., Kwon S., Jun E.K., Kim A., Whang K.Y., Kim H., Oh S., Yoon B.S., You S. (2011). Nanog-induced dedifferentiation of p53-deficient mouse astrocytes into brain cancer stem-like cells. Biochem. Biophys. Res. Commun..

[8] Di Fiore R., Marcatti M., Drago-Ferrante R., D'Anneo A., Giuliano M., Carlisi D., de Blasio A., Querques F., Pastore L., Tesoriere G. (2014). Mutant p53 gain of function can be at the root of dedifferentiation of human osteosarcoma MG63 cells into 3AB-OS cancer stem cells. Bone.

[9] Prince M.E., Sivanandan R., Kaczorowski A., Wolf G.T., Kaplan M.J., Dalerba P., Weissman I.L., Clarke M.F., Ailles L.E. (2007). Identification of a subpopulation of cells with cancer stem cell properties in head and neck squamous cell carcinoma. Proc. Natl. Acad. Sci. USA.

[10] Krishnamurthy S., Dong Z., Vodopyanov D., Imai A., Helman J.I., Prince M.E., Wicha M.S., Nor J.E. (2010). Endothelial cell-initiated signaling promotes the survival and self-renewal of cancer stem cells. Cancer Res..

[11] Qiao B., He B., Cai J., Yang W. (2014). The expression profile of Oct4 and Sox2 in the carcinogenesis of oral mucosa. Int. J. Clin. Exp. Pathol..

[12] Modur V., Thomas-Robbins K., Rao K. (2015). HPV and CSC in HNSCC cisplatin resistance. Front. Biosci. (Elite Ed.).

[13] Phillips T.M., McBride W.H., Pajonk F. (2006). The response of CD24^−/low^/CD44^+^ breast cancer-initiating cells to radiation. J. Natl. Cancer Inst..

[14] Li X., Lewis M.T., Huang J., Gutierrez C., Osborne C.K., Wu M.F., Hilsenbeck S.G., Pavlick A., Zhang X., Chamness G.C. (2008). Intrinsic resistance of tumorigenic breast cancer cells to chemotherapy. J. Natl. Cancer Inst..

[15] Diehn M., Cho R.W., Lobo N.A., Kalisky T., Dorie M.J., Kulp A.N., Qian D., Lam J.S., Ailles L.E., Wong M. (2009). Association of reactive oxygen species levels and radioresistance in cancer stem cells. Nature.

[16] Creighton C.J., Li X., Landis M., Dixon J.M., Neumeister V.M., Sjolund A., Rimm D.L., Wong H., Rodriguez A., Herschkowitz J.I. (2009). Residual breast cancers after conventional therapy display mesenchymal as well as tumor-initiating features. Proc. Natl. Acad. Sci. USA.

[17] Lagadec C., Vlashi E., Della Donna L., Meng Y., Dekmezian C., Kim K., Pajonk F. (2010). Survival and self-renewing capacity of breast cancer initiating cells during fractionated radiation treatment. Breast Cancer Res..

[18] Chen Y.C., Chen Y.W., Hsu H.S., Tseng L.M., Huang P.I., Lu K.H., Chen D.T., Tai L.K., Yung M.C., Chang S.C. (2009). Aldehyde dehydrogenase 1 is a putative marker for cancer stem cells in head and neck squamous cancer. Biochem. Biophys. Res. Commun..

[19] Facompre N., Nakagawa H., Herlyn M., Basu D. (2012). Stem-like cells and therapy resistance in squamous cell carcinomas. Adv. Pharmacol..

[20] Alison M.R., Guppy N.J., Lim S.M., Nicholson L.J. (2010). Finding cancer stem cells: are aldehyde dehydrogenases fit for purpose?. J. Pathol..

[21] Yu C.C., Hu F.W., Yu C.H., Chou M.Y. (2014). Targeting CD133 in the enhancement of chemosensitivity in oral squamous cell carcinoma-derived side population cancer stem cells. Head Neck.

[22] Fukusumi T., Ishii H., Konno M., Yasui T., Nakahara S., Takenaka Y., Yamamoto Y., Nishikawa S., Kano Y., Ogawa H. (2014). CD10 as a novel marker of therapeutic resistance and cancer stem cells in head and neck squamous cell carcinoma. Br. J. Cancer.

[23] Koukourakis M.I., Giatromanolaki A., Tsakmaki V., Danielidis V., Sivridis E. (2012). Cancer stem cell phenotype relates to radio-chemotherapy outcome in locally advanced squamous cell head-neck cancer. Br. J. Cancer.

[24] La Rocca G., Anzalone R., Corrao S., Magno F., Loria T., Lo Iacono M., Di Stefano A., Giannuzzi P., Marasa L., Cappello F. (2009). Isolation and characterization of Oct-4+/HLA-G+ mesenchymal stem cells from human umbilical cord matrix: Differentiation potential and detection of new markers. Histochem. Cell Biol..

[25] Marcus A.J., Woodbury D. (2008). Fetal stem cells from extra-embryonic tissues: Do not discard. J. Cell Mol. Med..

[26] Martens-de Kemp S.R., Brink A., Stigter-van Walsum M., Damen J.M., Rustenburg F., Wu T., van Wieringen W.N., Schuurhuis G.J., Braakhuis B.J., Slijper M. (2013). CD98 marks a subpopulation of head and neck squamous cell carcinoma cells with stem cell properties. Stem Cell Res..

[27] Arzumanyan A., Friedman T., Ng I.O., Clayton M.M., Lian Z., Feitelson M.A. (2011). Does the hepatitis B antigen HBx promote the appearance of liver cancer stem cells?. Cancer Res..

[28] Lindsey S., Langhans S.A. (2014). Crosstalk of oncogenic signaling pathways during epithelial-mesenchymal transition. Front. Oncol..

[29] Lin T., Chao C., Saito S., Mazur S.J., Murphy M.E., Appella E., Xu Y. (2005). p53 induces differentiation of mouse embryonic stem cells by suppressing Nanog expression. Nat. Cell Biol..

[30] Rangarajan A., Talora C., Okuyama R., Nicolas M., Mammucari C., Oh H., Aster J.C., Krishna S., Metzger D., Chambon P. (2001). Notch signaling is a direct determinant of keratinocyte growth arrest and entry into differentiation. EMBO J..

[31] Lowell S., Jones P., Le Roux I., Dunne J., Watt F.M. (2000). Stimulation of human epidermal differentiation by delta-notch signalling at the boundaries of stem-cell clusters. Curr. Biol..

[32] Talora C., Sgroi D.C., Crum C.P., Dotto G.P. (2002). Specific down-modulation of Notch1 signaling in cervical cancer cells is required for sustained HPV-E6/E7 expression and late steps of malignant transformation. Genes Dev..

[33] Gray G.E., Mann R.S., Mitsiadis E., Henrique D., Carcangiu M.L., Banks A., Leiman J., Ward D., Ish-Horowitz D., Artavanis-Tsakonas S. (1999). Human ligands of the Notch receptor. Am. J. Pathol..

[34] Looijenga L.H., Stoop H., de Leeuw H.P., de Gouveia Brazao C.A., Gillis A.J., van Roozendaal K.E., van Zoelen E.J., Weber R.F., Wolffenbuttel K.P., van Dekken H. (2003). POU5F1 (OCT3/4) identifies cells with pluripotent potential in human germ cell tumors. Cancer Res..

[35] Kim R.J., Nam J.S. (2011). OCT4 Expression enhances features of cancer stem cells in a mouse model of breast cancer. Lab. Anim. Res..

[36] Atlasi Y., Mowla S.J., Ziaee S.A., Bahrami A.R. (2007). OCT-4, an embryonic stem cell marker, is highly expressed in bladder cancer. Int. J. Cancer.

[37] Chang C.C., Shieh G.S., Wu P., Lin C.C., Shiau A.L., Wu C.L. (2008). Oct-3/4 expression reflects tumor progression and regulates motility of bladder cancer cells. Cancer Res..

[38] Masood R., Hochstim C., Cervenka B., Zu S., Baniwal S.K., Patel V., Kobielak A., Sinha U.K. (2013). A novel orthotopic mouse model of head and neck cancer and lymph node metastasis. Oncogenesis.

[39] Jamieson C.H., Ailles L.E., Dylla S.J., Muijtjens M., Jones C., Zehnder J.L., Gotlib J., Li K., Manz M.G., Keating A. (2004). Granulocyte-macrophage progenitors as candidate leukemic stem cells in blast-crisis CML. N. Engl. J. Med..

[40] Lapidot T., Sirard C., Vormoor J., Murdoch B., Hoang T., Caceres-Cortes J., Minden M., Paterson B., Caligiuri M.A., Dick J.E. (1994). A cell initiating human acute myeloid leukaemia after transplantation into SCID mice. Nature.

[41] Miyamoto T., Weissman I.L., Akashi K. (2000). AML1/ETO-expressing nonleukemic stem cells in acute myelogenous leukemia with 8;21 chromosomal translocation. Proc. Natl. Acad. Sci. USA.

[42] Al-Hajj M., Wicha M.S., Benito-Hernandez A., Morrison S.J., Clarke M.F. (2003). Prospective identification of tumorigenic breast cancer cells. Proc. Natl. Acad. Sci. USA.

[43] Hemmati H.D., Nakano I., Lazareff J.A., Masterman-Smith M., Geschwind D.H., Bronner-Fraser M., Kornblum H.I. (2003). Cancerous stem cells can arise from pediatric brain tumors. Proc. Natl. Acad. Sci. USA.

[44] Ignatova T.N., Kukekov V.G., Laywell E.D., Suslov O.N., Vrionis F.D., Steindler D.A. (2002). Human cortical glial tumors contain neural stem-like cells expressing astroglial and neuronal markers *in vitro*. GLIA.

[45] Singh S.K., Clarke I.D., Terasaki M., Bonn V.E., Hawkins C., Squire J., Dirks P.B. (2003). Identification of a cancer stem cell in human brain tumors. Cancer Res..

[46] Singh S.K., Hawkins C., Clarke I.D., Squire J.A., Bayani J., Hide T., Henkelman R.M., Cusimano M.D., Dirks P.B. (2004). Identification of human brain tumour initiating cells. Nature.

[47] Tang A.L., Owen J.H., Hauff S.J., Park J.J., Papagerakis S., Bradford C.R., Carey T.E., Prince M.E. (2013). Head and neck cancer stem cells: the effect of HPV—An *in vitro* and mouse study. Otolaryngol. Head Neck Surg..

[48] Mack B., Gires O. (2008). CD44s and CD44v6 expression in head and neck epithelia. PLoS ONE.

[49] Clay M.R., Tabor M., Owen J.H., Carey T.E., Bradford C.R., Wolf G.T., Wicha M.S., Prince M.E. (2010). Single-marker identification of head and neck squamous cell carcinoma cancer stem cells with aldehyde dehydrogenase. Head Neck.

[50] Sedghizadeh P.P., Billington W.D., Paxton D., Ebeed R., Mahabady S., Clark G.T., Enciso R. (2016). Is p16-positive oropharyngeal squamous cell carcinoma associated with favorable prognosis? A systematic review and meta-analysis. Oral Oncol..

[51] Salazar C.R., Smith R.V., Garg M.K., Haigentz M., Schiff B.A., Kawachi N., Anayannis N., Belbin T.J., Prystowsky M.B., Burk R.D. (2014). Human papillomavirus-associated head and neck squamous cell carcinoma survival: a comparison by tumor site and initial treatment. Head Neck Pathol..

[52] Zhang M., Kumar B., Piao L., Xie X., Schmitt A., Arradaza N., Cippola M., Old M., Agrawal A., Ozer E. (2014). Elevated intrinsic cancer stem cell population in human papillomavirus-associated head and neck squamous cell carcinoma. Cancer.

[53] Lee S.H., Lee C.R., Rigas N.K., Kim R.H., Kang M.K., Park N.H., Shin K.H. (2015). Human papillomavirus 16 (HPV16) enhances tumor growth and cancer stemness of HPV-negative oral/oropharyngeal squamous cell carcinoma cells via miR-181 regulation. Papillomavirus Res..

[54] Qian X., Wagner S., Ma C., Klussmann J.P., Hummel M., Kaufmann A.M., Albers A.E. (2013). ALDH1-positive cancer stem-like cells are enriched in nodal metastases of oropharyngeal squamous cell carcinoma independent of HPV status. Oncol. Rep..

[55] Iacovides D., Michael S., Achilleos C., Strati K. (2013). Shared mechanisms in stemness and carcinogenesis: lessons from oncogenic viruses. Front. Cell. Infect. Microbiol..

[56] Farrell P.J. (2002). Tumour viruses—Could they be an Achilles’ heel of cancer?. Eur. J. Cancer.

[57] Jin L., Xu Z.X. (2015). Recent advances in the study of HPV-associated carcinogenesis. Virol. Sin..

[58] Wise-Draper T.M., Wells S.I. (2008). Papillomavirus E6 and E7 proteins and their cellular targets. Front. Biosci..

[59] Chiche A., Moumen M., Petit V., Jonkers J., Medina D., Deugnier M.A., Faraldo M.M., Glukhova M.A. (2013). Somatic loss of p53 leads to stem/progenitor cell amplification in both mammary epithelial compartments, basal and luminal. Stem Cells.

[60] Dyson N., Howley P.M., Munger K., Harlow E. (1989). The human papilloma virus-16 E7 oncoprotein is able to bind to the retinoblastoma gene product. Science.

[61] O'Connor M.D., Wederell E., Robertson G., Delaney A., Morozova O., Poon S.S., Yap D., Fee J., Zhao Y., McDonald H. (2011). Retinoblastoma-binding proteins 4 and 9 are important for human pluripotent stem cell maintenance. Exp. Hematol..

[62] Cottage A., Dowen S., Roberts I., Pett M., Coleman N., Stanley M. (2001). Early genetic events in HPV immortalised keratinocytes. Genes Chromosomes Cancer.

[63] McLaughlin-Drubin M.E., Crum C.P., Munger K. (2011). Human papillomavirus E7 oncoprotein induces KDM6A and KDM6B histone demethylase expression and causes epigenetic reprogramming. Proc. Natl. Acad. Sci. USA.

[64] Zagouras P., Stifani S., Blaumueller C.M., Carcangiu M.L., Artavanis-Tsakonas S. (1995). Alterations in Notch signaling in neoplastic lesions of the human cervix. Proc. Natl. Acad. Sci. USA.

[65] Daniel B., Rangarajan A., Mukherjee G., Vallikad E., Krishna S. (1997). The link between integration and expression of human papillomavirus type 16 genomes and cellular changes in the evolution of cervical intraepithelial neoplastic lesions. J. Gen. Virol..

